# Genetic Characterization and Clinical Features of *Helicobacter pylori* Negative Gastric Mucosa-Associated Lymphoid Tissue Lymphoma

**DOI:** 10.3390/cancers13122993

**Published:** 2021-06-15

**Authors:** Barbara Kiesewetter, Christiane Copie-Bergman, Michael Levy, Fangtian Wu, Jehan Dupuis, Caroline Barau, Luca Arcaini, Marco Paulli, Marco Lucioni, Arturo Bonometti, Antonio Salar, Concepción Fernández-Rodriguez, Miguel A. Piris, Francesco Cucco, Rachel Dobson, Yan Li, Zi Chen, Cyrielle Robe, Ingrid Simonitsch-Klupp, Andrew Wotherspoon, Markus Raderer, Ming Qing Du

**Affiliations:** 1 Department of Pathology, Division of Cellular and Molecular Pathology, University of Cambridge, Cambridge CB2 1TN, UK; wftwendy@163.com (F.W.); fc437@cam.ac.uk (F.C.); rd571@cam.ac.uk (R.D.); liyan98_win@163.com (Y.L.); zc298@cam.ac.uk (Z.C.); 2 Department of Medicine I, Division of Oncology, Medical University of Vienna, 1090 Vienna, Austria; markus.raderer@meduniwien.ac.at; 3 Groupe Henri Mondor-Albert Chenevier, Département de Pathologie, APHP, INSERM U955, Université Paris Est, 94010 Créteil, France; christiane.copie@praxea-diagnostics.com (C.C.-B.); cyrielle.robe-ext@aphp.fr (C.R.); 4 Groupe Henri Mondor-Albert Chenevier and EC2M3 EA7375 Research Unit, Department of Gastroenterology, APHP, 94010 Créteil, France; michael.levy2@aphp.fr; 5 Department of Hematology, Pukou CLL Center, the First Affiliated Hospital of Nanjing Medical University, Nanjing 210029, China; 6 Lymphoid Malignancies Unit, APHP, Groupe Hospitalier Henri Mondor-Albert Chenevier, 94010 Créteil, France; jehan.dupuis@aphp.fr; 7 Plateforme de Ressources Biologiques BB-0033-00021, APHP, Groupe Hospitalier Henri Mondor-Albert Chenevier, 94010 Créteil, France; caroline.barau@aphp.fr; 8 Division of Hematology, Fondazione IRCCS Policlinico San Matteo, 27100 Pavia, Italy; arcaini@unipv.it; 9 Department of Molecular Medicine, University of Pavia, 27100 Pavia, Italy; 10 Department of Pathology, Fondazione IRCCS Policlinico San Matteo, 27100 Pavia, Italy; m.paulli@smatteo.pv.it (M.P.); m.lucioni@smatteo.pv.it (M.L.); 11 Anatomic Pathology, Department of Molecular Medicine University of Pavia & Fondazione IRCCS Policlinico San Matteo, 27100 Pavia, Italy; a.bonometti@smatteo.pv.it; 12 Department of Hematology, Hospital del Mar & Instituto de investigaciones médicas Hospital del Mar (IMIM), 08003 Barcelona, Spain; asalar@parcdesalutmar.cat; 13 Laboratory of Molecular Biology, Department of Pathology, Hospital del Mar, 08003 Barcelona, Spain; conchifernandezrodriguez@gmail.com; 14 Pathology Department, Hospital Fundación Jiménez Díaz, 28040 Madrid, Spain; miguel.piris@quironsalud.es; 15 Department of Haematology, Hebei General Hospital, Shijiazhuang 050000, China; 16 Department of Pathology, Medical University of Vienna, 1090 Vienna, Austria; ingrid.simonitsch-klupp@meduniwien.ac.at; 17 Department of Histopathology, The Royal Marsden Hospital, 203 Fulham Rd, London SW3 6JJ, UK; andrew.wotherspoon@rmh.nhs.uk

**Keywords:** extranodal lymphoma 1, MALT lymphoma 2, Helicobacter pylori 3, NF-κB pathway 4

## Abstract

**Simple Summary:**

The pathogenesis of *H. pylori*-associated gastric MALT lymphoma has been well characterized, but the genetic basis and clinical features of *H. pylori* negative gastric cases remain elusive. In the present study, we investigated the genetic profiles of a large series of *H. pylori* negative gastric MALT lymphoma by targeted sequencing for a panel of genes specifically designed for marginal zone lymphoma, together with assessment of common translocations and comprehensive clinical data. Targeted sequencing confirmed that NF-κB activation is a major driver in the pathogenesis of *H. pylori* negative MALT lymphoma, as shown by frequent *TNFAIP3* inactivating mutations and also by translocations of *MALT1/IGH*. This study adds new insights into the genetic background of *H. pylori* negative MALT lymphoma and will potentially allow us to more specifically target the underlying molecular pathways in future therapeutic concepts.

**Abstract:**

Background: In Western countries, the prevalence of gastric mucosa-associated lymphoid tissue (MALT) lymphoma has declined over the last three decades. Contemporaneously, *H. pylori* negative gastric MALT lymphoma is increasingly encountered, and their genetic basis and clinical features remain elusive. Methods: A total of 57 cases of *H. pylori* negative gastric MALT lymphoma were reviewed and investigated for chromosome translocation by fluorescence in-situ hybridization and for somatic mutations by the targeted sequencing of 93 genes. Results: *MALT1* translocation, most likely t(11;18)(q21;q21)/*BIRC3-MALT1,* was detected in 39% (22/57) cases, and *IGH* translocation was further seen in 12 *MALT1*-negative cases, together accounting for 60% of the cohort. Targeted sequencing was successful in 35 cases, and showed frequent mutations in NF-κB signaling pathways (*TNFAIP3* = 23%, *CARD11* = 9%, *MAP3K14* = 9%), together affecting 14 cases (40%). The NF-κB pathway mutations were mutually exclusive from *MALT1*, albeit not *IGH* translocation, altogether occurring in 86% of cases. There was no significant correlation between the genetic changes and clinicopathological parameters. The patients showed a median of progression-free survival (PFS) of 66.3 months, and a significant superior PFS when treated with systemic versus antibiotic therapy (*p* = 0.004). Conclusion: *H. pylori* negative gastric MALT lymphoma is characterized by highly frequent genetic changes in the NF-κB signaling pathways.

## 1. Introduction

Extranodal mucosa-associated lymphoid tissue lymphoma (MALT lymphoma) is a distinct type of indolent B-cell lymphoma with an age-adjusted incidence rate of 1.1/100.000 [1]. Histologically, the disease is characterized by a heterogeneous small B-cell infiltrate commonly showing lymphoepithelial lesions or follicular colonization and a typical marginal zone B-cell immunophenotype of CD20+CD5-CD10-cyclinD1- +/− light chain restriction [2,3]. While MALT lymphoma may arise in mucosa-associated tissues throughout the entire body, it is most prominent for its gastric manifestation strongly associated with chronic *H. pylori* gastritis [4,5]. Chronic *H. pylori* infection results in an active proinflammatory microenvironment, which facilitates clonal B-cell proliferation triggered by *H. pylori* specific T-cells through CD40-CD40L interaction and persistent cytokine release [3,6]. The causative role *of H. pylori* infection in gastric MALT lymphoma development was suggested by a high association between the lymphoma and *H. pylori* infection in epidemiologic studies, subsequently confirmed by a high efficacy of long-term lymphoma remissions in up to 80% of patients following a single course of antibiotics [7,8,9,10]. However, according to recent reports, there is an increasing trend of *H. pylori* negative gastric MALT lymphomas, accounting for 10–30% of cases [4,11,12,13]. Pathogenesis of these cases is scarcely understood at the moment, with no standard treatment defined so far [4,11,13]. 

At a molecular level, activation of the nuclear factor (NF) Kappa (κ) B pathway was considered as a central mechanism for MALT lymphoma, irrespective of their origin as shown by frequent genetic aberrations affecting the NF-kB pathway, including t(11;18)(q21;q21)/*BIRC3(API2)-MALT1*, t(14;18)(q32;q21)/*IGH-MALT1*, and t(1;14)(p22;q32)/*BCL10-IGH* [14]. This is further supported by findings of recurrent somatic mutations in the NF-κB/B-cell receptor (BCR) signaling molecules, including *TNFAIP3 (A20)*, *CARD11*, *CD79B*, and *MYD88.* There is growing evidence suggesting site-specific mutation profiles with variable involvement of the same genetic alterations at different anatomic sites [3,14]. For example, ocular adnexal MALT lymphoma features frequent mutations of *TNFAIP3* and *MYD88*, while those from the thyroid commonly showed *TNFRSF14* and *TET2* mutations [15]. In addition, whole exome sequencing of salivary gland and thyroid MALT lymphoma has revealed novel recurrent somatic mutations in G-protein coupled receptors (*GPR34* and *CCR6*) [16]. Finally, these NF-κB pathway mutations and *MALT1* translocations appeared to be enriched in gastric MALT lymphoma unresponsive to *H. pylori* eradication and were largely mutually exclusive, albeit based on a small series of cases [17]. 

Previous studies have shown that t(11;18)(q21;q21) and nuclear BCL10 overexpression/translocation are frequent events in *H. pylori* negative gastric MALT lymphoma, more common than in *H. pylori* positive cases [13,18]. However, to the best of our knowledge, there are currently no data on the molecular landscape of *H. pylori* negative gastric MALT lymphoma. In the present study, we investigated the genetic profiles of a large series of *H. pylori* negative gastric MALT lymphoma by targeted sequencing for a panel of genes (*n* = 93) specifically designed for marginal zone lymphoma, together with analysis of the MALT lymphoma associated translocations. In addition, we present comprehensive clinical data of 57 patients with *H. pylori* negative MALT lymphoma, which is the largest series investigated so far.

## 2. Materials and Methods

**Case selection and materials.** Formalin-fixed paraffin embedded (FFPE) diagnostic tissue biopsies and clinical data from a total of 57 patients with *H. pylori* negative gastric MALT lymphoma were identified from five European lymphoma centers (University Hospital Henry Mondor Créteil, Medical University of Vienna, Hospital del Mar Barcelona, University of Pavia, University Hospital Ramón y Cajal Madrid). All diagnoses were histologically verified according to the recent WHO classification of tumors of hematopoietic and lymphoid tissues [2]. FFPE gastric biopsy specimens at the initial diagnosis prior to anti-lymphoma treatment were available in each case. *H. pylori* negativity was confirmed by the absence of both *H. pylori* infection and evidence of *H. pylori* gastritis on histological examination, and a further negative result by polymerase chain reaction (PCR) for *H. pylori* DNA using high molecular weight DNA samples or a serological test for *H. pylori*-IgG, a urea breath test or a *H. pylori* stool antigen test before initiation of anti-lymphoma treatment. In all patients, clinical data were collected from routine medical records, including basic patient characteristics such as age, performance status and sex, stage of disease according to Lugano staging system, and risk profiles based on the MALT-IPI score [19]. In addition, treatment related data were collected, i.e., type of first line treatment; response based on radiological criteria, classified as complete remission (CR), partial remission (PR), stable disease (SD) and progressive disease (PD); and histological criteria (GELA), classified as CR, probable minimal residual disease (pMRD), responding residual disease (rRD), and no change (NC) [20]; as well as long-term outcome to calculate progression-free survival (PFS) and overall survival (OS).The study was performed in accordance with local ethical guidelines for the research use of tissue materials and clinical data. All further tissue-based investigations and data analysis were performed at the Department of Pathology, University of Cambridge, UK. 

**DNA extraction and quality assessment.** Hematoxylin and eosin (H and E) stained slides were reviewed to identify confluent lymphoma area with >30% tumor cells. Lymphoma enriched areas in each specimen were isolated by crude microdissection on consecutive tissue sections. DNA was extracted using the QIAamp DNA Micro Kit (QIAGEN, Crawly, Manchester, UK), quantified with a Qubit^®^ Fluorometer (Life Technologies, ThermoFisher Scientific, Waltham MA, USA) and assessed for quality by PCR of variably sized genomic fragments using a standardized protocol, as previously described [21].

**Targeted sequencing using HaloPlexHS enrichment and Illumina platform.** This was carried out for a previously published panel of 93 genes, which were specifically designed for marginal zone lymphoma and included those known to be recurrently mutated, and candidate genes based on our ongoing research and literature search [22]. Briefly, this includes genes involved in the NF-κB pathway, BCR signaling, B-cell development, and NOTCH signaling, but also genes involved in DNA damage/apoptosis/repair, epigenetic or translational regulators, and a broad panel of G-protein coupled receptors (GPCR) genes, including chemokine and adhesion receptors. HaloPlexHS target enrichment was performed according to manufacturer’s instructions for FFPE samples. This essentially included digestion of genomic DNA with restriction enzymes, followed by hybridization to the customized HaloPlexHS probes, ligation and circulation with HS DNA ligase, and purification and PCR-based amplification. The amplified library underwent final purification with AMPure XP beads (Beckman Coulter, Pasadena, CA, USA), was quantified using the 4200 TapeStation (Agilent Technologies, Santa Clara, CA, USA), and pooled. Sequencing was performed with the Illumina HiSeq4000 platform according to the manufacturer’s instructions. As in our previous study, DNA samples that were amenable for PCR of ≥400 bp genomic fragment were investigated in a single replicate, while those amplifiable at 300 bp were analyzed in duplicate [21]. 

**Sequence data analysis.** These were carried out using protocols and pipelines established in our recent studies, with single nucleotide variants (SNV) called using UnifiedGenotyper and additionally MuTect2 for variants at low variant allele frequency (VAF), while indels were identified using Pindel v0.2.5. [16,21]. Variants were filtered for read depth (excluded if <50), alternative allele depth (AAD) (excluded if <20), VAF (excluded if <2%), and SNPs with a minor allele frequency ≥ 0.1% and benign changes. Finally, the resulting variants were manually checked using the IGV software (Integrative Genomics Viewer). For samples with adequate DNA quality, variants that passed the above established filtering criteria were considered as a true change, while for those investigated in duplicate, only variants detected in both replicates were accepted.

**Florescence in situ hybridization (FISH).** FISH analysis was used to investigate translocation at the *MALT1* and *IGH* loci. As t(11;18)(q21;q21)/*BIRC3 (API2)-MALT1*, t(14;18)(q32;q21)/*IGH-MALT1* and t(1;14)(p22;q32)/*BCL10-IGH* are mutually exclusive, all samples were first investigated with a *MALT1* break-apart probe, and only negative cases were further analyzed with an *IGH* break apart probe (Abbott/Vysis, Abbott Park, IL, USA). 

**Statistical analysis.** IBM Statistics for Mac OS Version 26 (IBM, Armonk, NY, USA) was used for statistical analyses of data. Metric data were reported by median, mean, interquartile range (IQR), and absolute numbers for minimum/maximum. Absolute frequencies and corresponding percentages were presented for categorical variables. Qui-squared and Fisher’s exact test were used to test for associations of categorical variables. PFS and OS estimations were plotted with the Kaplan–Meier method and groups compared by log-rank test. Medians and 95% confidence intervals (CI) are reported; *p*-values < 0.05 were accepted as significant (two-sided).

## 3. Results

### 3.1. Clinical Characteristics

Among the 57 patients with *H. pylori* negative MALT lymphoma investigated in this study, median age at initial diagnosis was 61.8 years (range 36–84; IQR 53.5–72.4), and there were slightly more female patients (53% female versus 47% male). *H. pylori* negativity was diagnosed by histological assessment in 100% (57/57) of cases and further confirmed by PCR for *H. pylori* DNA from gastric biopsies in 56% (32/57), serology in 35% (20/57), breath test in 7% (4/57), and stool antigen test in 4% (2/57). Altogether, *H. pylori* negativity was confirmed by two or more different methods in 91% (52/57) of cases, with the remaining 5 cases showing no evidence of *H. pylori*/inflammation by histology. According to initial staging, 60% (34/57) of patients presented with localized MALT lymphoma, i.e., Lugano Stage I, 18% (10/57) had local lymph node involvement, i.e., Lugano stage II, and 23% (13/57) had distant lymph node or further extranodal involvement, i.e., Lugano stage IV. Regarding comorbidities, chronic hepatitis B or C virus was detected in two patients (2/57, 4%) and an autoimmune disorder was detected in two patients (2/57, 4%). Prognostic groups per MALT-IPI factors stratified for low risk in 47% (27/57), intermediate risk in 47% (27/57), and high risk in 5% (3/57) of patients. Table 1 shows a detailed list of clinical characteristics.

### 3.2. Chromsomal Translocation

All cases were investigated for *MALT1* chromosomal translocation by interphase FISH, and a *MALT1* rearrangement was detected in 39% (22/57) of cases. The rearrangement is most likely due to t(11;18)(q21;q21)/*BIRC3-MALT1* as t(14;18)/*IGH-MALT1* is rarely seen in gastric MALT lymphoma [23]. As translocation in MALT lymphoma is mutually exclusive, additional investigations with *IGH* break apart probes were performed only in *MALT1* negative cases, with the exception of 6 cases lacking sufficient material. *IGH* rearrangement was detected in a further 12 cases, likely due to t(1;14)(p22;q32)/*IGH-BCL10* as this is the most recurrent *IGH* involving translocation seen in gastric MALT lymphoma [14].

### 3.3. Somatic Mutations Detected in H. pylori Negative MALT Lymphoma

**Figure 1:**
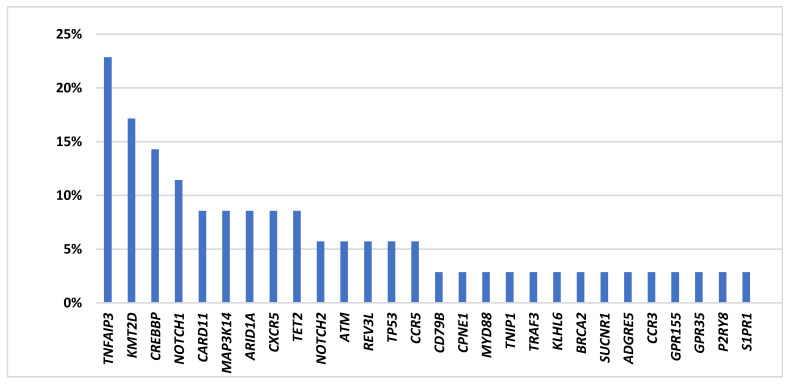
Frequencies of mutations detected by targeted sequencing in *H. pylori* negative gastric MALT lymphoma (*n* = 35).

**Targeted sequencing results.** Of the 57 cases included in the study, 39 had DNA samples meeting the minimal quality requirement for targeted sequencing using HaloPlexHS enrichment. Thirty-five of these cases were successfully investigated, while the remaining four cases failed to yield a sufficient library for sequencing. Among the 35 cases successfully sequenced with the Illumina HiSeq4000 platform, mean coverage was 94% (range 81.3–99.5%), with >90% coverage in 82% (29/35). In total, 67 potentially pathogenic variants were identified in 80% (28/35) of cases (mean number of variants per patient = 1.9; range, 0–6). In 7 cases, no pathogenic mutations were found within the gene panel. Detected variants affected 28 different genes including five genes showing double mutations (Figure 1, Supplementary Table S1). 

**Figure 2:**
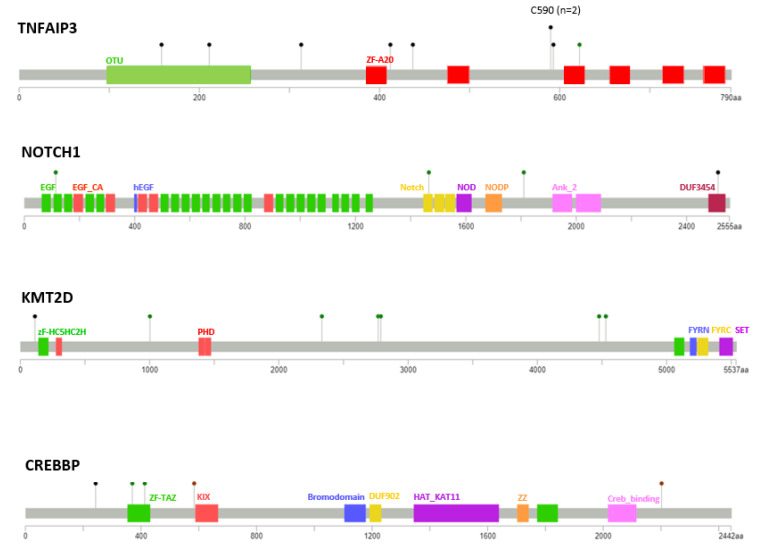
Distribution and characteristics of *TNFAIP3*, *NOTCH1*, *KMT2D*, and *CREBBP* mutations seen in *H. pylori* negative gastric MALT lymphoma. Black dots indicate truncating mutations, green dots missense mutations, and brown dots non-frameshift insertions/deletions. Functional domains are indicated once per gene in the respective color.

**Figure 3:**
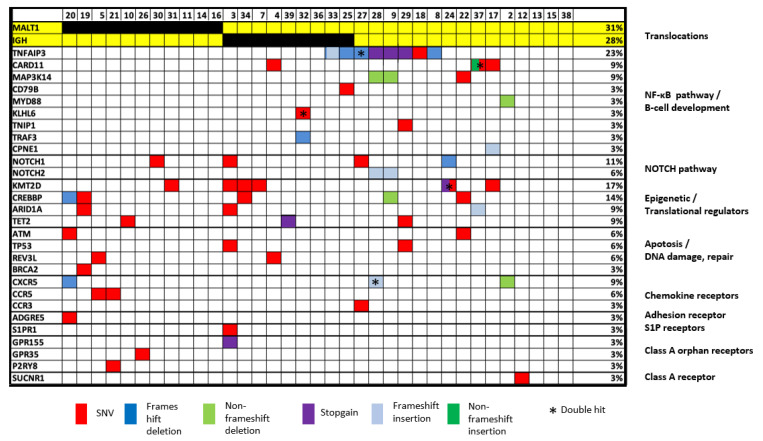
Heatmap illustration of genetic changes according to molecular pathways. Only cases successfully investigated by targeted sequencing are included in this figure (*n* = 35).

***TNFAIP3* is the most frequently mutated gene in *H. pylori* negative gastric MALT lymphoma.***TNFAIP3*, a negative regulator of NF-κB, was the most frequently affected gene, with mutations detected in 23% (8/35) of cases, including one case carrying two mutations (both frameshift deletions) (Figure 1, Figure 2 and Figure 3). *TNFAIP3* mutations included frameshift deletions in four cases, nonsense change in three cases and non-synonymous SNV and frameshift insertion each in one case. Apart from *TNFAIP3*, mutation was also frequently seen in several other genes that involve NF-κB signaling. These included *CARD11* (3/35 = 9%); *MAP3K14* (3/35 = 9%); and *CD79B*, *MYD88*, *TNIP1*, *CPNE1*, and *TRAF3* each in one patient (1/35 = 3%), respectively. Together, 40% (14/35) of cases showed one or more mutations in the NF-κB signaling pathways. 

Previous study of marginal zone lymphoma showed NOTCH signaling alterations, and we detected frequent *NOTCH1* (4/35 = 11%) and *NOTCH2* mutations (2/35 = 6%). Further pathways frequently altered involved epigenetic/translational regulator genes affecting *KMT2D* (6/35 = 17%), *CREBBP* (5/35 = 14%), *ARID1A* (3/35 = 9%), and *TET2* (3/35 = 9%); genes involved in apoptosis/DNA damage including *ATM* (2/35 = 6%) and *TP53* (2/35 = 6%); and chemokine receptor genes including *CXCR5* (3/35 = 9%), *CCR3* (1/35 = 3%), and *CCR5* (2/35 = 6%) (Figure 1, Figure 2 and Figure 3). Most mutations found had been previously described in MALT lymphoma, while the recently detected alterations in G-protein coupled receptors, reported previously by our group, were only detected at low frequency in *H. pylori* negative gastric MALT lymphoma (*GPR* 155, *n* = 1; *GPR* 35, *n* = 1) [16]. 

**Correlation among genetic changes.** *TNFAIP3* mutation was mutually exclusive from *MALT1* translocation, and also largely seen in those negative for *IGH* translocation (Figure 3). In addition, none of the 11 patients with a *MALT1* translocation showed any mutation in the NF-κB genes investigated (Figure 3). In contrast, 58% (14/24) of cases lacking *MALT1* translocation had one or more mutations in the NF-κB signaling pathways (*p* = 0.002). Similarly, combined analysis of patients with either *MALT1* or *IGH* rearrangement as one group showed a significant association between *MAP3K14* mutation and translocation negative cases (*p* = 0.04), and also a similar trend for *TNFAIP3* mutations (*p* = 0.07).

**Correlation of clinical characteristics and somatic genetic changes.** Fisher’s Exact tests for genetic alterations and univariate correlation with predefined clinical factors including disease dissemination status, gender, MALT-IPI scores, LDH, and age (>70 years) were investigated, but there was no apparent association between mutation and clinical characteristics. The only significant correlation was detected for stage of disease and *CXCR5*, with *CXCR5* mutations reported in 3% (1/30) of localized disease versus 40% (2/5) of disseminated disease (*p* = 0.047). Additionally, a non-significant trend was seen for *CREBBP*, with *CREBBP* mutations documented in 10% (3/30) of localized patients versus 40% (2/5) in disseminated patients (*p* = 0.14). In contrast, *TNFAIP3* mutation was exclusively detected in patients with localized disease (8/30, 25%), with no mutation in those with disseminated disease. Nonetheless, this did not reach statistical significance (27% versus 0%, *p* = 0.32). There was also no correlation between *MALT1* and *IGH* translocation and clinical features, although there was a trend towards a higher frequency of *MALT1* translocation in disseminated than localized cases (58% versus 33%, *p* = 0.18). Finally, we combined all mutations in the NF-κB signaling pathways and correlated with clinical characteristics; again, no significant association was found.

### 3.4. Response to Treatment and Long-Term Outcome

The median follow-up time for all 57 patients with clinical data available was 78.4 months (IQR 45–112). Regarding the first line treatment, 77% (44/57) of patients received up-front systemic therapy consisting of chemo and/or immunotherapy, 16% (9/57) were treated with *H. pylori* eradication antibiotics, and one patient each (2%, 1/57) received antiviral treatment for chronic hepatitis and radiotherapy. Rituximab (R) was a part of the systemic treatment in 35 patients (R-chemo-/immunotherapy, *n* = 22, R-monotherapy, *n* = 13), and the most frequently applied chemotherapy was chlorambucil +/−R in 20 patients (Table 2). Median time to first line treatment was 4.9 months (IQR 1.2–6.4 months). Two patients with stage I disease (4%, 2/57) had no active therapy but were closely managed by regular surveillance with endoscopies. Overall response (ORR) rate in the actively treated group was 75% (41/55), with 29% (16/41) achieving a CR and 46% (25/55) a PR (including histological responding residual disease and probable minimal residual disease according to GELA criteria), while stable disease was observed in 26% (14/55) and no primary progressive case was reported. The ORR was significantly higher for patients treated with systemic therapy with 84% (37/44) ORR for chemo-/ immunotherapy versus 22% (2/9) ORR in patients receiving antibiotics (*p* = 0.001). Time to best response was median 6 months (IQR 5.3–11.3 months).

**Figure 4:**
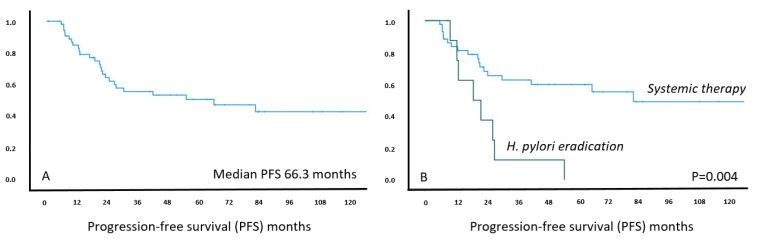
Kaplan–Meier curve for estimated progression-free survival in *H. pylori* negative MALT lymphoma patients (*n* = 57) (left panel **A**) and treated with systemic treatment versus *H. pylori* eradication (right panel **B**), respectively. X-axis: follow-up in months; Y-axis: cumulative progression-free survival.

After the first line therapy, 46% (26/57) showed disease progression during follow-up and 40% (23/57) received at least one further treatment. The median PFS for the entire cohort was over five years, being 66.3 months (95%CI 5.4–127.2). The number of cases showing relapses was much lower in the systemic than antibiotics treatment group (43% versus 77%, *p* = 0.76). In keeping with this, the estimated PFS was significantly longer in patients receiving systemic treatment than those treated with antibiotics (median PFS 82.7 months, 95%CI 26.5–138.9 versus 19.2 months, 95%CI 6.9–31.5) (*p* = 0.004, Figure 4).

**Figure 5:**
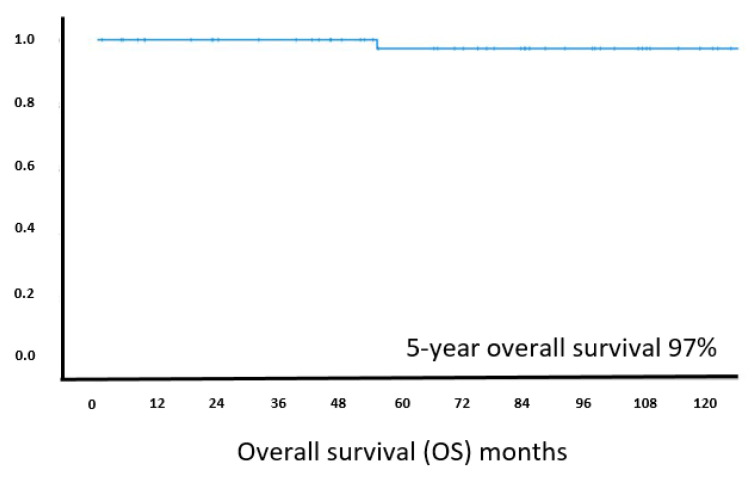
Kaplan–Meier curve for estimated overall survival in *H. pylori* negative MALT lymphoma patients (*n* = 57). X-axis: follow-up in months, Y-axis: cumulative overall survival.

Overall survival was excellent, with 97% of patients reported alive at 5 years follow-up and the median OS clearly not reached (Figure 5). At the last documented follow-up, 68% (39/57) of patients were alive without lymphoma, 30% (17/57) were alive with lymphoma, while one patient had died unrelated to lymphoma. Exploratory analysis of genetic results and long-term outcome revealed that neither a distinct somatic genetic change, nor the combined genetic changes in the NF-κB signaling pathways affect PFS or response to treatment. There was no significant difference in PFS between cases with and without a MALT1 translocation. However, there was a non-significant trend towards a worse PFS in patients with a mutation in *KMTD2* in comparison with those without the mutation by log-rank test (*p* = 0.125) and similarly in patients with a *MAP3K14* mutation versus wild type status (*p* = 0.18).

Finally, we also performed an exploratory analysis of the largest treatment subgroup, i.e., patients receiving systemic therapy, but again no influence of *MALT1* translocation or NF-κB signaling mutations regarding ORR or PFS were detected.

## 4. Discussion

While initially all gastric MALT lymphomas were thought to be associated with *H. pylori*, there is mounting evidence showing an increase of *H. pylori* negative gastric MALT lymphoma, reported variably in up to 30% of cases at tertiary referral centers [11,12]. The reason for this remains elusive, but it is of importance to ascertain true *H. pylori* negativity, as an absence of *H. pylori* solely based on histological examination does not constitute definitive proof of negativity, because proton pump inhibitor-intake, chronic atrophic gastritis, and intestinal metaplasia may mask occult *H. pylori* infection [8]. In the current manuscript, we present a large series of well-characterized *H. pylori* negative MALT lymphomas and their genetic characterization by targeted sequencing of a panel of genes specifically designed for marginal zone lymphoma.

Our study confirmed that the alterations in the NF-κB signaling pathway constitute the central player in MALT lymphoma development, also for *H. pylori* negative MALT lymphoma [14]. A high proportion (39%) of the *H. pylori* negative gastric MALT lymphoma had a *MALT1* rearrangement, most likely due to t(11;18)(q21;q21)/*BIRC3-MALT1*. *IGH* rearrangements, again likely due to t(1;14)(p22;q32)/*BLC10-IGH*, were documented in 24% of cases investigated. Targeted sequencing revealed mutations in the NF-κB pathway in 40% of cases, exclusively in those lacking *MALT1* translocation, with *TNFAIP3* mutation being the most frequent change (23%). In addition, potential activating mutations were seen in *CARD11*, a gene involved in canonical NF-κB activation through formation of the so-called CBM complex (CARD11-BCL10-MALT1), and *MAP3K14*, which acts via non-canonical NF-κB pathways (both in 9%) [14]. In total, 86% of patients had at least one potentially pathogenic mutation in the NF-κB signaling pathways, including not only canonical but also potentially non-canonical activation [24]. 

Other molecular pathways affected by mutation include NOTCH signaling (17%), epigenetic and translational regulators (40%), genes involved in apoptosis and DNA damage repair (20%), and chemokine receptors (17%). The frequency of *TNFAIP3*, *TET2*, and *NOTCH1* mutations in *H. pylori* negative gastric MALT lymphoma is similar to those seen in unselected gastric MALT lymphomas, whereas the previously reported *TNFRSF14* alterations affecting gastric MALT lymphomas were absent in the current series [16]. Interestingly, G-protein coupled receptors, which had previously been detected by our group at high frequency for salivary and thyroid MALT lymphoma, were rare (*GPR155, n* = 1; *GPR* 35, *n* = 1) in *H. pylori* negative gastric MALT lymphoma, further reinforcing the concept of site-specific mutation profiles as previously suggested [16]. 

A previous study investigated 19 cases of *H. pylori* gastric MALT lymphoma unresponsive to antibiotic therapy by sequencing a panel of 425 genes [17]. These authors reported a frequency of *TNFAIP3* (16%) and *NOTCH1* (16%) mutation, similar to those seen in the present study, and *TRAF3* alterations (21%), which were absent in our cases. Similarly to our findings, they reported *TNFAIP3* mutations and *TRAF3* mutations to be mutually exclusive from *MALT1* translocation. In view of this, one might speculate that *H. pylori* negative MALT lymphoma and *H. pylori* positive MALT lymphoma that are resistant to eradication may share a common genetic profile. 

One limitation of such retrospective analyses is the lack of sufficient material in some cases for targeted sequencing. This is a well-known difficulty in investigating gastric MALT lymphoma with often too little material available due to the endoscopic diagnostic approach and frequently macroscopically occult lesions. Nonetheless, by careful microdissection to enrich tumor cells and assessment of DNA quality, we were able to generate a good quality of sequence data in 61% of cases by targeted sequencing using archival diagnostic tissue specimens with many years of storage [21]. 

The basic clinical features of our 57 patients correspond well to MALT lymphoma cohorts previously presented in terms of median age, female-to-male ratio, and stage of disease [4]. One feature reported notably low in the current cohort is, however, the presence of autoimmune disorders; while 30–40% of patients had been diagnosed with an additional autoimmune disorder in another series reported in the literature [25], only 2/57 patients had a documented autoimmune condition in our analysis. Whereas the absolute number does not allow for correlation of this feature with somatic mutation data, this suggests that autoimmune disorders might not be involved in *H. pylori* negative MALT lymphoma.

In terms of treatment-related data including long-term and short-term outcome, the current series is the largest cohort of *H. pylori* negative gastric MALT lymphoma with stringent diagnostic criteria for *H. pylori* negativity. There are no established guidelines for treatment of *H. pylori* negative MALT lymphoma. *H. pylori* eradication has been shown to be effective at variable frequency; radiotherapy for localized disease or chemo-immunotherapy can be used, with the latter two resulting in high response rates but considerable toxicity in some cases [8,9,26]. In our cohort, 77% of patients received up-front systemic treatment, which was chemotherapy-based in the majority of cases and resulted in an objective response rate of 75% and no primary progressive disease. The PFS of the entire cohort was long, at 66.3 months, but systemic therapy was superior to *H. pylori* eradication (83 versus 19 months, *p* = 0.004). Most patients treated with systemic therapy received R +/− chlorambucil, and results were in line with published data for gastric MALT lymphoma; however, no difference in ORR or PFS regarding *MALT1* status (as previously suggested) was observed [27,28]. In the literature, responses to *H. pylori* eradication were reported in up to 30–40% in small series and a recent meta-analysis, and these effects may be partly contributed to the immunomodulatory effects of the macrolide antibiotic clarithromycin commonly used for eradication [29,30,31,32,33]. Effects of antibiotics were particularly observed in patients with localized disease [34,35]. In addition, *Helicobacter* species other than *H. pylori* have been suspected to explain the antibiotic treatment responses [36,37]. In the present study, nine *H. pylori* negative patients were treated with antibiotics (stage I, *n* = 6; stage II, *n* = 2; stage IV, *n* = 1). While the response rate of 22% and the PFS of 19 months for antibiotics only in our cohort appears reasonable, the lower long-term efficacy in comparison with the chemo-/immune collective probably also underlines the “true” negativity of *H. pylori* in our cases. PFS was comparable if only stage I patients were analyzed (median PFS 22.5 months). One might hypothesize that false negative cases may contribute to eradication responders in some publications, while in our cohort a strict two step algorithm was applied to confirm *H. pylori* negativity. Nevertheless, none of our patients died due to lymphoma or experienced transformation during follow-up.

## 5. Conclusions

In the present study, we again confirm that NF-κB activation is a major driver in the pathogenesis of *H. pylori* negative MALT lymphoma, as shown by highly frequent translocations and *TNFAIP3* inactivating mutations. In view of this and the fact that true *H. pylori* negative MALT lymphoma often appear to be antibiotic non-responders, it would be of interest to further pursue therapeutic strategies of NF-κB inhibition. Inhibition of MALT1 protease activity is a potential treatment approach of interest, and proof of concept data are available for several compounds [38,39,40,41]; however, there are no clinical relevance yet for MALT lymphoma. One already established drug in this context is bortezomib, a proteasome inhibitor dampening NF-κB activities, which resulted in a high response rate of up to 80% for MALT lymphoma but was not further followed due to hemato- and neurotoxicity [42,43]. In view of our data and new advances in development of next generation proteasome inhibitors, further investigation with a focus on *H. pylori* negative MALT lymphoma might be interesting.

## Figures and Tables

**Table 1:** Clinical features of 57 patients with *H. pylori* negative gastric MALT lymphoma.

Clinical Characteristics	Entire Collective (*n* = 57)	NGS Collective (*n* = 35/57)
Sex (female/ male)	52.6% (30/57)/47.4% (27/57)	51.4% (18/35)/48.6% (17/35)
Age (median)	61.8 years (range 36–84)	65.7 years (range 36–80)
***H. pylori* negativity confirmed by**		
Histology	100% (57/57)	100% (35/35)
PCR for H. pylori	56.1% (32/57)	48.6% (17/35)
Serology	35.1% (20/57)	42.9% (15/35)
Breath test	7% (4/57)	2.9% (1/35)
Stool antigen test	3.5% (2/57)	-
**Lugano staging system**		
Lugano stage I	59.6% (34/57)	62.9% (22/35)
Lugano stage II	17.5% (10/57)	20% (7/35)
Lugano stage IV	22.8% (13/57)	17.1% (6/35)
**MALT IPI status**		
Low risk	47.4% (27/57)	51.4% (18/35)
Intermediate risk	47.4% (27/57)	45.7% (16/35)
High risk	5.3% (3/57	2.9% (1/57)
**Further clinical features**		
Autoimmune disorder	3.5% (2/57)	5.7% (2/35)
LDH > upper normal limit	5.3% (3/57)	5.7% (2/35)
Hepatitis B/C virus	3.5% (2/57)	none
**Translocation status**		
MALT1 rearrangement	38.6% (22/57)	31.4% (11/35)
IGH rearrangement	23.5% (12/51)	28.1% (9/32)
**First line treatment**		
Chemo-/immunotherapy	77.2% (44/57)	71.4% (25/35)
Antibiotics (=eradication)	15.8% (9/57)	22.9% (8/35)
Watch and wait	3.5% (2/57)	5.7% (2/35)
Radiotherapy	1.8% (1/57)	-
Antiviral therapy hepatitis	1.8% (1/57	-
Median follow-up time (IQR)	78.4 months (44.9–112)	66.5 months (23.9–98.3)

Abbreviations in chronological order: *H*. = *Helicobacter*; PCR = polymerase chain reaction; MALT = mucosa-associated lymphoid tissue; NGS = next generation sequencing, IPI = international prognostic index; LDH = lactate dehydrogenase levels, IQR = interquartile range.

**Table 2:** First line treatment and outcome of 57 patients with *H. pylori* negative MALT lymphoma.

Treatment/Response	Outcome
Entire Collective (*n* = 57)	
Complete remission	28.1% (16/57)
Partial remission	43.9% (25/57)
Stable disease	28.1% (16/57)
PFS (median) months	66.3 (95%CI 5.4–127.2)
Actively treated collective * (*n* = 55)	
Complete remission	29.1% (16/55)
Partial remission	45.5% (25/55)
Stable disease	25.5% (14/55)
PFS (median) months	66.3 (95%CI 5.4–127.2)
Chemo-/immunotherapy (*n* = 44) **	
Complete remission	31.8% (14/44)
Partial remission	52.3% (23/44)
Stable disease	15.9% (7/44)
PFS (median) months	82.7 (95%CI 26.5–138.9)
**Antibiotics (HP eradication) (*n* = 9)**	
Complete remission	None
Partial remission	22.2% (2/9)
Stable disease	77.8% (7/9)
PFS (median) months	19.2 (95%CI 6.9–31.5)
**Watch and wait (*n* = 2)**	
Stable disease	100% (2/2)
PFS (absolute) months	5.1 and 0.9
**Radiotherapy (*n* = 1)**	
Complete remission	100% (1/1)
PFS (absolute) months	84.0
**Antiviral therapy hepatitis (*n* = 1)**	
Complete remission	100% (1/1)
PFS (absolute) months	105.2

* excluding watch and wait, *n* = 2. ** includes: chlorambucil +/− rituximab (R), *n* = 20; R-monotherapy, *n* = 13; lenalidomide +/− R *n* = 2, R-CHOP, *n* = 2; R-CVP, *n* = 2; R-bendamustine, *n* = 2; ofatumumab, *n* = 1; R-cyclophosphamide, *n* = 1; azithromycin, *n* = 1. Abbreviations in chronological order: H. = *Helicobacter*; MALT = mucosa-associated lymphoid tissue; PFS = progression-free survival, *HP* = *Helicobacter pylori*.

## Data Availability

The data presented in this study are available in Table S1.

## Supplementary Materials

The following are available online at https://www.mdpi.com/article/10.3390/cancers13122993/s1: Table S1: Complete list of mutations detected by next generation sequencing (*n* = 67).
