# Supplementary material for: Genetic Characterization and Clinical Features of Helicobacter pylori Negative Gastric Mucosa-Associated Lymphoid Tissue Lymphoma
Source: Cancers (Basel). 2021 Jun 15;13(12):2993. doi: 10.3390/cancers13122993 (PMC8232676; doi:10.3390/cancers13122993)
Supplement: Supplementary file 1 [file cancers-13-02993-s001.zip › cancers-1245918-supplementary.pdf]

cancers MDPI

Supplementary Table 1. Complete list of mutations detected by next generation sequencing (n=67). 1

SampleID	Variant.ID	Gene	AAChange.refGene
HP_GA_3	chr1:101239997_G>T	***S1PR1***	S1PR1:NM_001400:exon2:c.G1013T:p.G338V
HP_GA_3	chr1:26766309_A>G	***ARID1A***	ARID1A:NM_006015:exon9:c.A2821G:p.I941V
HP_GA_3	chr12:49030976_C>T	***KMT2D***	KMT2D:NM_003482:exon40:c.G13588A:p.D4530N
HP_GA_17	chr12:49039223_G>A	***KMT2D***	KMT2D:NM_003482:exon33:c.C8365T:p.R2789W
HP_GA_29	chr4:105275192_C>T	***TET2***	TET2:NM_001127208:exon11:c.C4682T:p.S1561F
HP_GA_4	chr6:111357108_G>A	***REV3L***	REV3L:NM_002912:exon19:c.C7090T:p.P2364S
HP_GA_24	chr12:49031272_C>T	***KMT2D***	KMT2D:NM_003482:exon39:c.G13433A:p.R4478Q
HP_GA_19	chr13:32341069_T>G	***BRCA2***	BRCA2:NM_000059:exon11:c.T6714G:p.D2238E
HP_GA_27	chr3:46265586_G>A	***CCR3***	CCR3:NM_178329:exon2:c.G428A:p.R143Q
HP_GA_5	chr6:111367821_A>T	***REV3L***	REV3L:NM_002912:exon15:c.T5967A:p.D1989E
HP_GA_30	chr9:136502043_C>G	***NOTCH1***	NOTCH1:NM_017617:exon29:c.G5430C:p.Q1810H
HP_GA_27	chr9:136505498_C>A	***NOTCH1***	NOTCH1:NM_017617:exon25:c.G4398T:p.Q1466H
HP_GA_19	chr1:26761013_G>A	***ARID1A***	ARID1A:NM_006015:exon5:c.G2078A:p.R693Q
HP_GA_25	chr17:63929447_T>A	***CD79B***	CD79B:NM_000626:exon5:c.A578T:p.D193V
HP_GA_26	chr2:240630508_G>A	***GPR35***	GPR35:NM_005301:exon2:c.G556A:p.V186M
HP_GA_12	chr3:151881106_G>C	***SUCNR1***	SUCNR1:NM_033050:exon3:c.G563C:p.S188T
HP_GA_4	chr7:2945828_T>G	***CARD11***	CARD11:NM_032415:exon4:c.A349C:p.T117P
HP_GA_37	chr7:2945834_A>T	***CARD11***	CARD11:NM_032415:exon4:c.T343A:p.F115I
HP_GA_21	chrX:1466156_G>T	***P2RY8***	P2RY8:NM_178129:exon2:c.C403A:p.R135S
HP_GA_22	chr11:108256239_C>T	***ATM***	ATM:NM_000051:exon14:c.C2149T:p.R717W
HP_GA_7	chr12:49050580_G>A	***KMT2D***	KMT2D:NM_003482:exon11:c.C3008T:p.P1003L
HP_GA_10	chr4:105261759_G>A	***TET2***	TET2:NM_001127208:exon8:c.G3955A:p.E1319K
HP_GA_29	chr5:151063732_T>G	***TNIP1***	TNIP1:NM_006058:exon3:c.A152C:p.E51A

HP_GA_18	chr6:137879309_G>A	***TNFAIP3***	TNFAITNFAIP3:NM_006290:exon7:c.G1864A:p.G622S
HP_GA_17	chr7:2937980_T>A	***CARD11***	CARD11:NM_032415:exon8:c.A1070T:p.D357V
HP_GA_3	chr9:136523780_C>T	***NOTCH1***	NOTCH1:NM_017617:exon3:c.G340A:p.G114R
HP_GA_22	chr17:45290495_G>C	***MAP3K14***	MAP3K14:NM_003954:exon2:c.C251G:p.A84G
HP_GA_32	chr3:183492118_G>A	***KLHL6***	KLHL6:NM_130446:exon7:c.C1675T:p.R559W
HP_GA_9	chr6:137879215_C>A	***TNFAIP3***	TNFAIP3:NM_006290:exon7:c.C1770A:p.C590X
HP_GA_28	chr6:137879215_C>A	***TNFAIP3***	TNFAIP3:NM_006290:exon7:c.C1770A:p.C590X
HP_GA_29	chr6:137879222_C>T	***TNFAIP3***	TNFAIP3:NM_006290:exon7:c.C1777T:p.Q593X
HP_GA_20	chr11:108289015_C>T	***ATM***	ATM:NM_000051:exon28:c.C4148T:p.S1383L
HP_GA_31	chr12:49039283_C>T	***KMT2D***	KMT2D:NM_003482:exon33:c.G8305A:p.D2769N
HP_GA_34	chr12:49040773_G>A	***KMT2D***	KMT2D:NM_003482:exon31:c.C6997T:p.P2333S
HP_GA_3	chr2:174460026_G>T	***GPR155***	GPR155:NM_152529:exon10:c.C1623A:p.C541X
HP_GA_32	chr3:183555469_A>T	***KLHL6***	KLHL6:NM_130446:exon1:c.T185A:p.L62Q
HP_GA_21	chr3:46373183_G>T	***CCR5***	CCR5:NM_000579:exon3:c.G281T:p.W94L
HP_GA_5	chr3:46373519_C>T	***CCR5***	CCR5:NM_000579:exon3:c.C617T:p.P206L
HP_GA_39	chr4:105272735_C>T	***TET2***	TET2:NM_001127208:exon10:c.C4354T:p.R1452X
HP_GA_9	chr1:119923937_->GGTCACAG	***NOTCH2***	NOTCH2:NM_024408:exon26:c.4558_4559insCTGTGACC:p.Q1520fs
HP_GA_28	chr1:119923937_->GGTCACAG	***NOTCH2***	NOTCH2:NM_024408:exon26:c.4558_4559insCTGTGACC:p.Q1520fs
HP_GA_37	chr1:26774925_->C	***ARID1A***	ARID1A:NM_006015:exon18:c.4699dupC:p.R1566fs
HP_GA_28	chr11:118894590_->C	***CXCR5***	CXCR5:NM_001716:exon2:c.1047dupC:p.C349fs
HP_GA_20	chr11:118894608_GGCGCAGGAGC>-	***CXCR5***	CXCR5:NM_001716:exon2:c.1064_1074del:p.W355fs
HP_GA_28	chr11:118894626_->T	***CXCR5***	CXCR5:NM_001716:exon2:c.1083dupT:p.S361fs
HP_GA_2	chr11:118894628_GAGTCA>-	***CXCR5***	CXCR5:NM_001716:exon2:c.1084_1089del:p.362_363del
HP_GA_24	chr12:49054591_C>A	***KMT2D***	KMT2D:NM_003482:exon3:c.G337T:p.E113X
HP_GA_32	chr14:102886230_C>-	***TRAF3***	TRAF3:NM_145725:exon7:c.612delC:p.C204fs
HP_GA_9	chr16:3728427_TGCTGCTGCTGTTGC>-	***CREBBP***	CREBBP:NM_004380:exon31:c.6606_6620del:p.2202_2207del

HP_GA_22	chr16:3780803_AGA>-	***CREBBP***	CREBBP:NM_004380:exon8:c.1750_1752del:p.584_584del
HP_GA_19	chr16:3792073_C>T	***CREBBP***	CREBBP:NM_004380:exon5:c.G1238A:p.R413Q
HP_GA_34	chr16:3793494_G>C	***CREBBP***	CREBBP:NM_004380:exon4:c.C1108G:p.R370G
HP_GA_20	chr16:3850364_GTTA>-	***CREBBP***	CREBBP:NM_004380:exon2:c.728_731del:p.L243fs
HP_GA_9	chr17:45290490_ACTCAG>-	***MAP3K14***	MAP3K14:NM_003954:exon2:c.251_256del:p.84_86del
HP_GA_28	chr17:45290490_ACTCAG>-	***MAP3K14***	MAP3K14:NM_003954:exon2:c.251_256del:p.84_86del
HP_GA_29	chr17:7674199_A>C	**TP53**	TP53:NM_000546:exon7:c.T764G:p.I255S
HP_GA_3	chr17:7674220_C>T	**TP53**	TP53:NM_000546:exon7:c.G743A:p.R248Q
HP_GA_20	chr19:14390962_G>A	**ADGRE5**	ADGRE5:NM_078481:exon4:c.G229A:p.G77R
HP_GA_17	chr20:35627313_->T	**CPNE1**	CPNE1:NM_152925:exon14:c.1202_1203insA:p.F401fs
HP_GA_2	chr3:38141208_CACTGTCTGCGACTACACCAACCCCTG>-	**MYD88**	MYD88:NM_002468:exon5:c.852_878del:p.284_293del
HP_GA_25	chr6:137875023_CTATGATACTCGGGTAGGTTTTTCCCCCTAATTAT>-	**TNFAIP3**	TNFAIP3:NM_006290:exon3:c.474_486del:p.C158fs
HP_GA_33	chr6:137875833_->T	**TNFAIP3**	TNFAIP3:NM_006290:exon4:c.632_633insT:p.S211fs
HP_GA_27	chr6:137877209_CGTCCAAGGCTGGGACCATGGCA>-	**TNFAIP3**	TNFAIP3:NM_006290:exon6:c.939_961del:p.P313fs
HP_GA_27	chr6:137878680_A>-	**TNFAIP3**	TNFAIP3:NM_006290:exon7:c.1235delA:p.Q412fs
HP_GA_8	chr6:137878756_CT>-	**TNFAIP3**	TNFAIP3:NM_006290:exon7:c.1311_1312del:p.A437fs
HP_GA_37	chr7:2939887_->TCC	**CARD11**	CARD11:NM_032415:exon6:c.725_726insGGA:p.E242delinsEE
HP_GA_24	chr9:136496197_AG>-	**NOTCH1**	NOTCH1:NM_017617:exon34:c.7541_7542del:p.P2514fs
